# Granisetron for Postoperative Nausea and Vomiting Prophylaxis in Microvascular Decompression Despite Prominent Non-serotonergic Emetic Pathways

**DOI:** 10.7759/cureus.112568

**Published:** 2026-07-13

**Authors:** Hiroyuki Oishi, Takenori Kato, Nobuaki Hagiwara, Toshinori Hasegawa

**Affiliations:** 1 Neurosurgery, Komaki City Hospital, Komaki, JPN; 2 Anesthesiology, Komaki City Hospital, Komaki, JPN

**Keywords:** 5-ht3 receptor antagonist, granisetron, microvascular decompression (mvd), postoperative nausea and vomiting (ponv), serotonin receptor

## Abstract

Background

Microvascular decompression (MVD) is associated with a high incidence of postoperative nausea and vomiting (PONV). Unlike in general surgery, where PONV is predominantly serotonin-mediated, MVD additionally activates non-serotonergic emetic mechanisms, including vestibular stimulation and direct brainstem perturbation. Whether selective 5-HT₃ receptor antagonists can effectively prevent PONV in MVD remains unclear.

Materials and methods

This retrospective, exploratory, quasi-experimental before-after study included 40 consecutive MVD patients at a single institution, divided into a granisetron group (n = 20; 1 mg intravenously at dural closure) and a control group (n = 20) based on an institutional protocol change. Primary outcomes included PONV incidence, nausea severity, vomiting episodes, and rescue antiemetic use.

Results

Using the predefined moderate-to-severe threshold (nausea severity score ≥4, vomiting, or rescue antiemetic use), the 24-hour PONV incidence was lower in the granisetron group than in the control group (35% vs. 80%), corresponding to an absolute risk reduction (ARR) of 45% and a relative risk reduction (RRR) of 56% (p = 0.010). At six hours, the granisetron group showed lower nausea severity scores (4.0 (0-6.0) vs. 8.5 (6.75-9.0); p = 0.002) and fewer vomiting episodes (0 (0-1.0) vs. 1 (0.75-2.0); p = 0.022). Early mobilization and oral intake were also improved. Because the analysis was exploratory and the sample size limited, the estimates should be interpreted as hypothesis-generating.

Conclusions

In this exploratory, single-center, quasi-experimental before-after study of 40 patients, prophylactic granisetron was associated with an RRR of 56% and an ARR of 45% (number needed to treat ≈ 2.2) for moderate-to-severe PONV following MVD, consistent with a substantial serotonergic contribution to PONV in this setting. The residual PONV rate of 35% suggests that non-serotonergic emetic mechanisms remain unaddressed. Given the small sample size and single-center design, these findings should be regarded as hypothesis-generating rather than confirmatory; nonetheless, they support the evaluation of multimodal antiemetic strategies and may inform the design of adequately powered future studies on optimal antiemetic strategies for MVD.

## Introduction

Postoperative nausea and vomiting (PONV) is a common complication of surgery and anesthesia, with an incidence of 20-30% in the general surgical population [1-3]. Among neurosurgical procedures, microvascular decompression (MVD) is associated with an especially high risk of PONV, with reported incidence rates of 60-70% [4]. This elevated risk has been attributed to the proximity of the surgical site to the brainstem emetic centers, vestibular stimulation during cerebellar retraction, and direct manipulation of the brainstem [1,3-6]. Importantly, these mechanisms involve both serotonergic and non-serotonergic neural pathways, which may have distinct implications for the efficacy of specific antiemetic agents.

The nucleus tractus solitarius (NTS) in the medulla serves as the final common pathway through which emetic inputs converge to trigger the vomiting reflex [7]. Three principal afferent pathways stimulate the NTS: (1) vagal afferents from the gastrointestinal tract, where enterochromaffin cells release serotonin (5-hydroxytryptamine (5-HT)) in response to surgical and anesthetic stress, activating 5-HT₃ receptors on vagal nerve terminals that project to the NTS; (2) the chemoreceptor trigger zone (CTZ) in the area postrema, which detects emetogenic substances in the blood and cerebrospinal fluid (CSF) through 5-HT₃ and other receptors and relays signals to the adjacent NTS; and (3) the vestibular system, which transmits signals to the NTS primarily via histamine H₁ and muscarinic receptors, with limited serotonergic involvement [7-9]. In general surgical procedures, where PONV is predominantly mediated through pathways 1 and 2, 5-HT₃ receptor antagonists have demonstrated consistent prophylactic efficacy [10].

In MVD, however, the surgical procedure activates emetic mechanisms beyond pathways 1 and 2. Cerebellar retraction and manipulation of the cerebellopontine angle stimulate the vestibular system (pathway 3), generating emetic signals through histaminergic and cholinergic mechanisms that are not primarily dependent on serotonin [1,3,6]. Moreover, the proximity of the surgical field to the brainstem emetic centers means that the CTZ, NTS, and vestibular nuclei can be directly stimulated through mechanical perturbation, independent of any receptor-mediated pathway. Because pathway 3 and these direct mechanical stimuli are not blocked by 5-HT₃ receptor antagonists, selective 5-HT₃ receptor blockade may be less effective in preventing PONV after MVD. Granisetron, a selective 5-HT₃ receptor antagonist approved for PONV prophylaxis in Japan in 2021, has demonstrated efficacy in various surgical populations [11,12]; however, there are insufficient data on its specific efficacy after MVD, in which non-serotonergic emetic mechanisms are particularly prominent.

This study had two objectives. The primary clinical objective was to evaluate the prophylactic efficacy of granisetron in reducing the incidence and severity of PONV in patients undergoing MVD for trigeminal neuralgia and hemifacial spasm. The secondary, exploratory mechanistic objective was to examine whether a 5-HT₃ receptor antagonist can provide a clinically meaningful reduction in PONV in a surgical setting where non-serotonergic emetic pathways are also significantly involved. By comparing the relative risk reduction (RRR) and absolute risk reduction (ARR) with those reported in general surgical populations, we sought to explore the relative contribution of serotonergic mechanisms to PONV in MVD and to provide a pathway-based rationale for targeted antiemetic strategies, with the intent of informing the design of future adequately powered studies.

## Materials and methods

Study design and patients

This exploratory, quasi-experimental before-after study analyzed all 40 consecutive patients who underwent MVD performed by a single, highly experienced neurosurgeon at Komaki City Hospital (Aichi, Japan) between January 2021 and October 2025, with no exclusions. The study included a complete cohort of MVD cases performed by the surgeon during this period. This design used an institutional protocol change as a natural intervention, comparing outcomes in patients treated before and after the introduction of granisetron prophylaxis; this quasi-experimental approach is recognized as appropriate when prospective randomization is not feasible. This study was approved by the Institutional Review Board of Komaki City Hospital (approval number: 251007), and the requirement for informed consent was waived because of the retrospective nature of the study.

In October 2024, our institution implemented prophylactic granisetron administration as standard care for MVD procedures. The patients were divided into two groups based on this protocol change: the granisetron group included 20 consecutive patients who underwent MVD from October 2024 onward, whereas the control group included 20 consecutive patients who underwent MVD immediately before September 2024. No premedication, including prophylactic antiemetics, atropine sulfate, narcotics, or sedatives, was administered before surgery. All procedures were performed under total intravenous anesthesia (TIVA) using propofol and remifentanil. No volatile anesthetics or nitrous oxide were used, as TIVA provides superior neurophysiological monitoring capabilities for MVD procedures. Intraoperative fentanyl and remifentanil were administered according to the anesthesiologist's instructions following standard protocols, with appropriate dose adjustments as needed. It should be noted that fentanyl use decreased after national supply restrictions began in December 2024 owing to manufacturing issues. In the granisetron group, 1 mg of granisetron was administered intravenously at the time of dural closure, in accordance with the standard dosing regimen approved by the National Health Insurance.

Operative procedure

All MVD procedures were performed using standardized techniques. The patients were positioned laterally with the head secured in a Sugita head frame (Mizuho Medical Co., Ltd., Tokyo, Japan), and a C-shaped skin incision was made behind the ear. All operations were performed under microscopic guidance with continuous auditory brainstem response (ABR) monitoring throughout the procedure, and abnormal muscle response (AMR) monitoring was additionally employed for patients with hemifacial spasm. ABR monitoring was used to detect potential neurological complications, with surgical manipulation temporarily halted when the wave V latency was prolonged by ≥1.0 ms and resumed when the latency returned to within 1 ms of baseline. Our surgical approach avoids brain retractors to minimize cerebellar retraction and tissue manipulation. In patients with trigeminal neuralgia, the horizontal fissure was opened to access the trigeminal nerve. In patients with hemifacial spasm, the procedure was completed upon confirmation of the disappearance of AMR [13]. In all cases, a transposition technique was employed to separate the offending vessel from the affected cranial nerve. Postoperative care, including analgesia (opioids were not routinely administered postoperatively) and nursing-assessed documentation of mobilization and oral intake, followed standardized institutional protocols that were applied uniformly across both study periods.

Data collection

Patient data, including demographic information (age and sex), surgical details (surgical time and diagnosis), and PONV risk factors, were collected from the electronic medical records. The Apfel score was calculated for each patient based on four risk factors: female sex, history of PONV or motion sickness, nonsmoking status, and postoperative opioid use [14]. Although the Apfel score includes postoperative opioid use as a risk factor, opioids were not routinely administered postoperatively in our neurosurgical procedures. In addition to the standard Apfel score, we recorded intraoperative fentanyl and remifentanil administration as additional background factors for analysis.

Outcome measures

Primary outcome measures included the incidence and severity of postoperative nausea, the number of vomiting episodes, and the frequency of rescue antiemetic medication use. Nausea severity was assessed using a numerical rating scale (NRS; 0-10, where 0 indicated no nausea and 10 indicated the worst possible nausea). Patients were asked to rate the intensity of their nausea at each assessment time point. Nursing staff recorded the occurrence of vomiting episodes and the use of rescue antiemetic medications. PONV was defined as the presence of any of the following within the first 24 hours postoperatively: nausea severity score ≥4 on the NRS (indicating moderate-to-severe nausea), occurrence of vomiting, or use of rescue antiemetic medication. This threshold was selected to capture clinically significant PONV that affects patient comfort, early mobilization, and recovery. A sensitivity analysis using a broader definition (nausea severity score ≥1) was also performed to assess the consistency of the treatment effect across different severity thresholds.

The secondary outcome measures included the level of mobilization and the volume of oral intake. Mobilization was evaluated using the Surgical Intensive Care Unit Optimal Mobilization Score (SOMS, 0-4), where 0 indicated complete bed rest, 1 the ability to sit up in bed, 2 the ability to sit at the bedside, 3 the ability to stand, and 4 the ability to walk [15]. Oral intake was recorded as the percentage of each meal consumed. All outcome measures were evaluated at six, 24, and 48 hours postoperatively. Rescue antiemetic medication (metoclopramide 10 mg) was administered intravenously when patients reported moderate-to-severe nausea (NRS ≥4) or experienced vomiting.

Statistical analysis

Data were analyzed using SPSS Statistics version 26.0 (IBM Corp. Released 2019. IBM SPSS Statistics for Windows, Version 26.0. Armonk, NY: IBM Corp.). Given the modest sample size, all continuous variables are presented as medians with interquartile ranges (IQRs) and were compared using the Mann-Whitney U test. Categorical variables are presented as frequencies and percentages and were compared using Fisher's exact test. Statistical significance was set at p < 0.05. Given the exploratory nature of this study, no adjustment for multiple comparisons was applied; all p-values are therefore reported as descriptive and hypothesis-generating rather than confirmatory. The ARR was calculated as the difference in PONV incidence between the granisetron and control groups, and the RRR was calculated as ARR divided by the control-group incidence. The 95% confidence interval (CI) for the ARR was calculated using the Wald method. CIs were derived only for the primary composite endpoint, and secondary outcomes are reported without CIs, in keeping with the exploratory nature of the analysis. No a priori sample-size calculation was performed; the sample size was determined by the number of consecutive cases available before and after the protocol change.

## Results

Patient characteristics

A total of 40 patients who underwent MVD were included in this study, with 20 in the granisetron group and 20 in the control group. The granisetron group consisted of 11 patients with trigeminal neuralgia and nine with hemifacial spasm, whereas the control group included nine patients with trigeminal neuralgia and 11 with hemifacial spasm. There were no significant differences between the two groups in median age (66 (54-76) vs. 62 (55-71) years; p = 0.784); operating time (204 (190-239) vs. 231 (213-260) min; p = 0.07); intraoperative remifentanil dose (2,143 (1,697-2,390) vs. 2,290 (1,571-2,910) μg; p = 0.517); or the distribution of primary diseases (p = 0.752). However, significant differences were observed in other characteristics: the granisetron group had a significantly lower intraoperative fentanyl dose (200 (100-300) vs. 300 (300-350) μg; p = 0.010) and higher Apfel scores (2 (2-3) vs. 2 (1-2); p = 0.016) than the control group (Table 1).

**Table 1:** Patient characteristics and surgical information Data are presented as the number of patients (n, %) or median (IQR); n = 20 per group. Female sex and hemifacial spasm represent the complementary proportions. Continuous variables were compared with the Mann-Whitney U test; categorical variables with Fisher's exact test. * p < 0.05, ** p < 0.01. IQR: interquartile range, OR: odds ratio, U: Mann-Whitney U statistic

Variable	Granisetron group (n = 20)	Control group (n = 20)	p-value	Test statistic
Age (years), median (IQR)	66 (54-76)	62 (55-71)	0.784	U = 180
Male sex, n (%)	7 (35%)	8 (40%)	0.925	OR = 0.81
Operating time (min), median (IQR)	204 (190-239)	231 (213-260)	0.07	U = 110
Intraoperative fentanyl (μg), median (IQR)	200 (100-300)	300 (300-350)	0.010**	U = 86
Intraoperative remifentanil (μg), median (IQR)	2,143 (1,697-2,390)	2,290 (1,571-2,910)	0.517	U = 148
Apfel score, median (IQR)	2 (2-3)	2 (1-2)	0.016*	U = 244
Trigeminal neuralgia, n (%)	11 (55%)	9 (45%)	0.752	OR = 1.49

Primary outcomes

Despite the significantly higher Apfel scores in the granisetron group, the primary outcome favored granisetron. The incidence of moderate-to-severe PONV within the first 24 hours (nausea severity score ≥4, vomiting, or rescue antiemetic use) was significantly lower in the granisetron group than in the control group (35% vs. 80%; ARR, 45%; 95% CI, 18-72%; RRR, 56%; p = 0.010). A sensitivity analysis using a broader threshold (nausea severity score ≥1) also showed a lower PONV incidence in the granisetron group (65% vs. 95%; ARR, 30%; 95% CI, 7-53%; RRR, 32%; p = 0.044), with the between-group difference consistent across severity thresholds.

The granisetron group experienced less severe nausea and fewer vomiting episodes than the control group during the early postoperative period. At six hours postoperatively, the median nausea NRS score was notably lower in the granisetron group (4.0 (0-6.0) vs. 8.5 (6.75-9.0); p = 0.002). At 24 hours postoperatively, the granisetron group continued to show lower nausea severity scores (1.0 (0-4.0) vs. 4.0 (0.75-6.0); p = 0.03). At 48 hours postoperatively, the granisetron group showed a lower value than the control group (0 (0-1.0) vs. 0 (0-4.0); p = 0.316), although this difference was not statistically significant.

The number of vomiting episodes within the first six hours was significantly lower in the granisetron group (0 (0-1.0) vs. 1.0 (0.75-2.0); p = 0.022). Between 6 and 24 hours postoperatively, both groups experienced minimal vomiting: 0 (0-0) in the granisetron group vs. 0 (0-0.25) in the control group (p = 0.565). Between 24 and 48 hours, no vomiting occurred in the granisetron group, whereas only minimal episodes occurred in the control group (0 (0-0) vs. 0 (0-0); p = 0.199). The frequency of rescue antiemetic use during the first six hours was also lower in the granisetron group (0 (0-1.0) vs. 1.0 (0.75-1.0); p = 0.168), although this difference did not reach statistical significance. No significant differences were observed in rescue antiemetic use after the first six postoperative hours (Table 2).

**Table 2:** PONV outcomes in the granisetron and control groups Data are presented as the number of patients (n, %) or median (IQR); n = 20 per group. Composite PONV was compared with Fisher's exact test; continuous variables with the Mann-Whitney U test. * p < 0.05, ** p < 0.01. IQR: interquartile range, NRS: numerical rating scale (0-10), OR: odds ratio, PONV: postoperative nausea and vomiting, U: Mann-Whitney U statistic

Variable	Granisetron group (n = 20)	Control group (n = 20)	p-value	Test statistic
Composite PONV, n (%)				
NRS ≥4, vomiting, or rescue antiemetic	7 (35%)	16 (80%)	0.010**	OR = 0.135
NRS ≥1, vomiting, or rescue antiemetic	13 (65%)	19 (95%)	0.044*	OR = 0.098
Nausea severity, NRS, median (IQR)				
6 h	4.0 (0-6.0)	8.5 (6.75-9.0)	0.002**	U = 68
24 h	1.0 (0-4.0)	4.0 (0.75-6.0)	0.03*	U = 100
48 h	0 (0-1.0)	0 (0-4.0)	0.316	U = 140
Vomiting episodes, median (IQR)				
0-6 h	0 (0-1.0)	1.0 (0.75-2.0)	0.022*	U = 99
6-24 h	0 (0-0)	0 (0-0.25)	0.565	U = 156
24-48 h	0 (0-0)	0 (0-0)	0.199	U = 153
Rescue antiemetic use, median (IQR)				
0-6 h	0 (0-1.0)	1.0 (0.75-1.0)	0.168	U = 128
6-24 h	0 (0-0)	0 (0-1.0)	0.251	U = 140
24-48 h	0 (0-0)	0 (0-0)	0.715	U = 154

Secondary outcomes

The level of mobilization measured by SOMS was significantly higher in the granisetron group at six hours postoperatively (2 (2-4) vs. 0 (0-1); p < 0.001), indicating better early mobilization. By 24 hours, both groups showed improved mobilization, with no significant difference between them (4 (3-4) vs. 3 (1-4); p = 0.126). At 48 hours, all patients in both groups achieved similar mobilization scores (4 (4-4) vs. 4 (3-4); p = 0.162). With respect to oral intake, the granisetron group demonstrated significantly higher intake at breakfast on postoperative day (POD) 1 (10.0 (0-55.0)% vs. 0 (0-0)%; p = 0.02). Although oral intake remained generally higher in the granisetron group throughout subsequent measurements, statistical significance was not reached at other time points, including at POD 2 dinner (100 (35.0-100)% vs. 55.0 (21.25-91.25)%; p = 0.061). There was no significant difference in the length of hospital stay between the granisetron and control groups (5 (5-9) vs. 7 (6-8) days; p = 0.12) (Table 3). No clinically apparent or documented adverse events related to granisetron administration were identified in the medical records during the study period.

**Table 3:** Secondary outcomes: mobilization, oral intake, and length of stay Data are presented as median (IQR). All comparisons were performed with the Mann-Whitney U test. * p < 0.05, *** p < 0.001. IQR: interquartile range, POD: postoperative day, SOMS: Surgical Intensive Care Unit Optimal Mobilization Score (0-4), U: Mann-Whitney U statistic

Variable	Granisetron group (n = 20)	Control group (n = 20)	p-value	Test statistic
Mobilization, SOMS (0-4), median (IQR)				
6 h	2 (2-4)	0 (0-1)	<0.001***	U = 306
24 h	4 (3-4)	3 (1-4)	0.126	U = 217
48 h	4 (4-4)	4 (3-4)	0.162	U = 205
Oral intake, % of meal consumed, median (IQR)				
POD 1 breakfast	10.0 (0-55.0)	0 (0-0)	0.02*	U = 237
POD 1 lunch	50.0 (5.0-75.0)	7.5 (0-76.25)	0.269	U = 206
POD 1 dinner	75.0 (50.0-100)	37.5 (10.0-100)	0.536	U = 190
POD 2 breakfast	90.0 (40.0-100)	65.0 (25.0-86.25)	0.194	U = 212
POD 2 lunch	85.0 (40.0-100)	57.5 (17.5-78.75)	0.134	U = 219
POD 2 dinner	100 (35.0-100)	55.0 (21.25-91.25)	0.061	U = 230
Length of hospital stay (days), median (IQR)	5 (5-9)	7 (6-8)	0.12	U = 120

## Discussion

In this exploratory study, prophylactic granisetron was associated with a lower incidence and severity of PONV following MVD. Using the moderate-to-severe threshold defined in the Materials and Methods, the PONV incidence within 24 hours was 35% in the granisetron group compared with 80% in the control group, yielding an ARR of 45% (p = 0.010) and an RRR of 56%. Even with a broader definition capturing all symptoms (nausea severity score ≥1), the ARR was 30% (65% vs. 95%; p = 0.044). The treatment effect was most pronounced in the early postoperative period (within six hours), with consistent differences across multiple outcome measures, including nausea severity, vomiting episodes, early mobilization, and oral intake.

We did not perform an a priori sample-size calculation, and the analysis was therefore exploratory; the sample size was determined by the number of consecutive cases available before and after the protocol change rather than by a predefined power target. Accordingly, the effect estimates should be interpreted as hypothesis-generating, and the relatively wide CI around the ARR (95% CI, 18-72%) reflects the limited precision inherent to a study of this size. We deliberately did not perform post hoc (observed) power calculations because power computed from the observed effect is a deterministic function of the p-value and provides no additional inferential information.

The 80% control-group incidence of moderate-to-severe PONV is higher than the 60-70% commonly reported in previous MVD studies [4]. This difference primarily reflects our definition of PONV. Previous studies have often used narrower criteria, such as moderate-to-severe symptoms or vomiting alone. In contrast, our composite threshold (defined in the Materials and Methods) provides a comprehensive assessment of clinically significant PONV. Notably, a sensitivity analysis using an even broader definition (nausea severity score ≥1) yielded a control-group incidence of 95%. Yet the between-group difference remained statistically significant, indicating that the effect direction was consistent regardless of the PONV definition used.

Baseline differences between the two groups warrant consideration. The granisetron group had significantly higher Apfel scores (p = 0.016), suggesting a theoretically greater PONV risk, and significantly lower intraoperative fentanyl doses (p = 0.010) owing to national supply restrictions. Validated PONV risk-prediction models such as the Apfel score address postoperative rather than intraoperative opioid use, and intraoperative opioids have generally not been found to contribute substantially to PONV [16,17]; some more recent data, however, suggest that higher intraoperative fentanyl doses may modestly increase PONV risk when other predictors are considered. Even under this more conservative assumption, intraoperative fentanyl is unlikely to explain our findings: it was the control group that received the higher fentanyl doses, so any such effect would act to increase-not decrease-PONV in the control group. Furthermore, the granisetron group carried the higher predicted PONV risk (higher Apfel scores) yet showed markedly lower moderate-to-severe PONV (35% vs. 80%; ARR, 45%; RRR, 56%), making it unlikely that these baseline differences materially account for the observed reduction; if anything, they render our estimate conservative.

**Figure 1:**
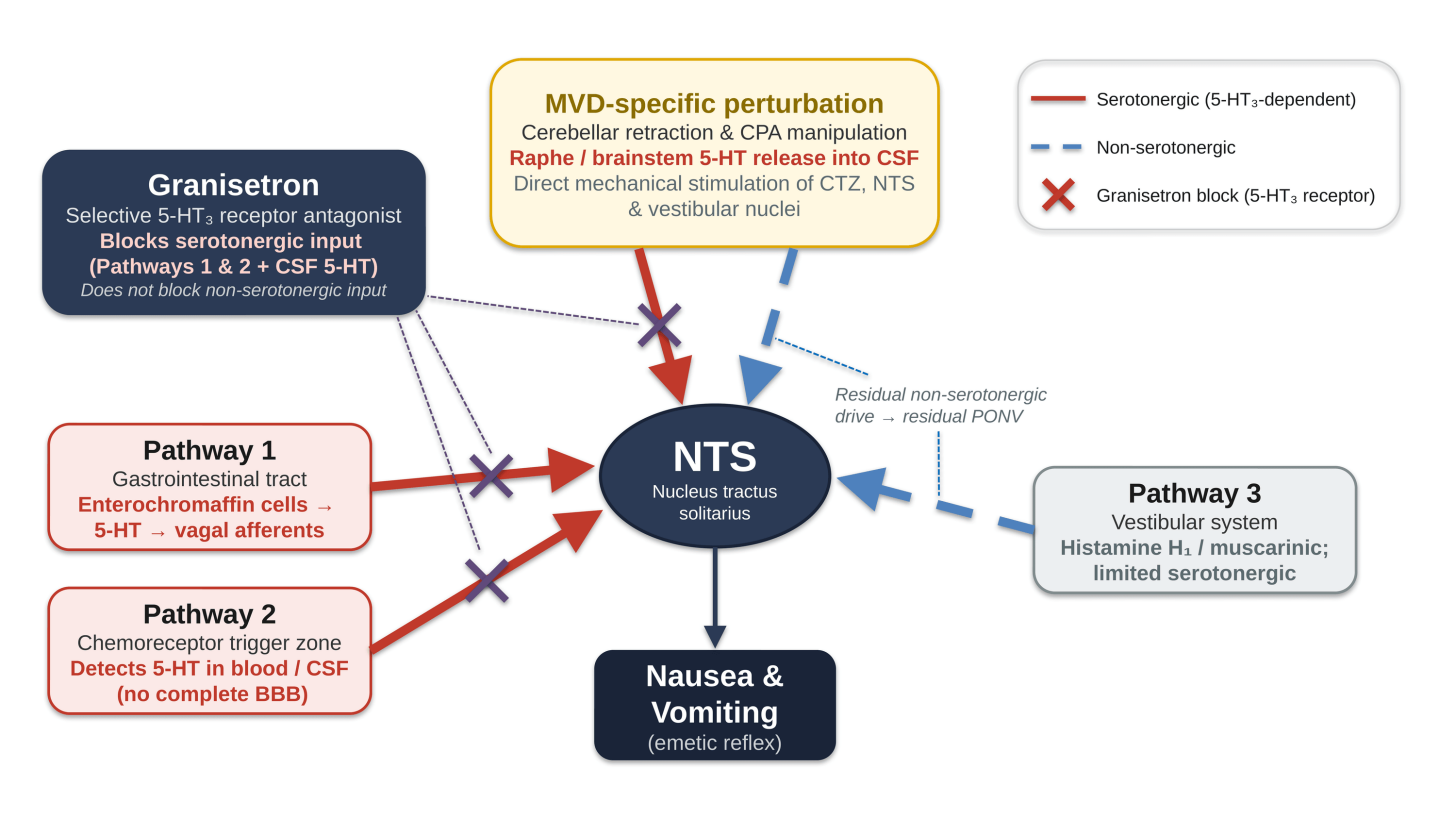
Convergent emetic pathways in MVD and the site of action of granisetron, a selective 5-HT₃ receptor antagonist Convergent emetic pathways in MVD and the site of action of granisetron. Three principal afferent pathways converge on the NTS: vagal afferents from the gastrointestinal tract (pathway 1) and the CTZ (pathway 2) are predominantly serotonergic and 5-HT₃-dependent (solid red), whereas the vestibular system (pathway 3) signals mainly through histamine H₁ and muscarinic receptors with limited serotonergic involvement (dashed blue). MVD additionally induces serotonin release from raphe/brainstem structures into the CSF and directly perturbs the CTZ, NTS, and vestibular nuclei. Granisetron, a selective 5-HT₃ receptor antagonist, blocks the serotonergic inputs (red crosses) but not the non-serotonergic inputs, which may account for the residual PONV observed despite prophylaxis. 5-HT: 5-hydroxytryptamine (serotonin), BBB: blood-brain barrier, CPA: cerebellopontine angle, CSF: cerebrospinal fluid, CTZ: chemoreceptor trigger zone, MVD: microvascular decompression, NTS: nucleus tractus solitarius, PONV: postoperative nausea and vomiting. Image Credit: Authors using PowerPoint (Microsoft Corporation, Redmond, WA, USA).

The emetic reflex is triggered through three principal afferent pathways that converge on the NTS (Figure 1) [7]. General anesthesia and surgical stress impair gastrointestinal motility and stimulate enterochromaffin cells in the small intestine to release serotonin, which activates 5-HT₃ receptors on vagal afferent terminals projecting to the NTS (pathway 1) [8,18]. Released serotonin also enters the circulation and stimulates 5-HT₃ receptors in the CTZ of the area postrema, which then relays signals to the adjacent NTS (pathway 2). The third pathway originates from the vestibular system, which transmits signals to the NTS primarily via histamine H₁ and muscarinic receptors, with limited serotonergic involvement [7,9]. In general surgical procedures, PONV is predominantly mediated through pathways 1 and 2, both of which are largely serotonergic; accordingly, 5-HT₃ receptor antagonists provide effective prophylaxis in these settings [10]. In MVD, however, pathways 1 and 2 are operative as in any surgery. Still, pathway 3 is also substantially activated because cerebellar retraction and manipulation of the cerebellopontine angle structures stimulate the vestibular nuclei [1,6]. Moreover, the proximity of the surgical field to the brainstem emetic centers means that the CTZ, NTS, and vestibular nuclei can be directly stimulated through mechanical perturbation, independent of any specific receptor-mediated pathway [3,19]. Because pathway 3 and these direct mechanical stimuli are not primarily dependent on serotonin signaling, it was theoretically conceivable that a selective 5-HT₃ receptor antagonist might provide insufficient PONV prophylaxis in MVD.

Despite these theoretical concerns, our results showed a clinically meaningful RRR of 56%, suggesting that serotonergic mechanisms play a substantial role in MVD-related PONV. A key mechanism that may explain this finding is the release of serotonin from brainstem structures during surgical manipulation. The raphe nuclei, located along the midline of the brainstem, are the primary source of serotonergic neurons in the central nervous system, and their caudal components project directly to the NTS [9,20]. Elevated CSF 5-HT levels have been identified as an independent risk factor for PONV within 24 hours after MVD [8], providing direct evidence that surgical manipulation in the cerebellopontine angle triggers serotonin release into the CSF. This released serotonin can stimulate 5-HT₃ receptors on both the CTZ, which lacks a complete blood-brain barrier, and the NTS, thereby activating the serotonergic emetic pathway even in a surgical setting where non-serotonergic stimuli are also prominent. Granisetron effectively blocks these receptors, which may partially account for the substantial reduction in PONV observed in our study. Thus, MVD may provide a unique clinical model in which serotonergic and non-serotonergic emetic pathways are simultaneously active, allowing indirect estimation of the relative contribution of each pathway through the response to a selective 5-HT₃ receptor antagonist. Because CSF serotonin was not measured in the present cohort, this mechanistic account remains a hypothesis and relies on external data.

The RRR of 56% observed in our study appears broadly comparable to the efficacy of 5-HT₃ receptor antagonists in general surgical populations. A comprehensive Cochrane network meta-analysis reported that granisetron monotherapy reduced the risk of postoperative vomiting with a risk ratio of approximately 0.59 compared with placebo, corresponding to an RRR of approximately 41% [10]. This is an indirect comparison across different populations, endpoints, and study designs and should therefore be regarded as hypothesis-generating rather than a formal efficacy comparison. However, despite granisetron prophylaxis, 35% of patients in the granisetron group still experienced moderate-to-severe PONV, a rate that remains higher than the baseline PONV incidence of 20-30% in general surgical populations [1-3]. This residual PONV likely reflects the contribution of non-serotonergic emetic mechanisms, particularly vestibular stimulation via pathway 3 and direct mechanical stimulation of brainstem emetic centers, neither of which is blocked by 5-HT₃ receptor antagonists.

An important clinical implication is that the high baseline incidence of PONV in MVD amplifies the absolute benefit of granisetron prophylaxis. The ARR of 45% translates to a number needed to treat (NNT) of approximately 2.2, meaning that for every two to three patients treated, one additional patient is spared moderate-to-severe PONV. This NNT is considerably more favorable than those typically reported for 5-HT₃ receptor antagonists in general surgical populations, where baseline PONV rates of 20-30% yield NNTs of 6-8 [18]. Thus, even though granisetron cannot fully address the non-serotonergic component of MVD-related PONV, its prophylactic administration appears to be of high clinical value in this population.

To address the residual PONV attributable to non-serotonergic mechanisms, a multimodal antiemetic strategy warrants consideration. Agents targeting the vestibular pathway, such as antihistamines or anticholinergics (e.g., scopolamine), may complement the serotonergic blockade provided by granisetron by acting on pathway 3. Additionally, neurokinin-1 receptor antagonists such as aprepitant offer broad-spectrum antiemetic activity across multiple emetic pathways and may be particularly suitable for MVD, where diverse mechanisms converge [10,18,21,22]. Dexamethasone, which acts through anti-inflammatory and possibly central mechanisms, also remains a valuable adjunct [23]. The gradual decrease in granisetron efficacy observed beyond six hours postoperatively in our study, consistent with its plasma half-life of approximately 10 hours [24], further supports the rationale for combining agents with complementary durations and mechanisms of action. These multimodal regimens are proposed as hypotheses to be tested in future prospective trials rather than as established, evidence-based standards of care for MVD at present.

The surgical techniques employed at our institution may have contributed to the observed efficacy of granisetron. Our MVD approach avoids the use of brain retractors, minimizing cerebellar retraction and potentially reducing both vestibular stimulation (pathway 3) and direct mechanical stress on brainstem emetic centers. Continuous ABR monitoring, with temporary cessation of manipulation when wave V latency was prolonged, helped limit excessive brainstem perturbation. These techniques may have attenuated non-serotonergic emetic stimuli, thereby enhancing the relative contribution of serotonergic pathways susceptible to granisetron blockade. This may partly explain why our results showed a more favorable response than previously reported for 5-HT₃ antagonist monotherapy in neurosurgical procedures [11,25-27]. Because these refined techniques, particularly the avoidance of brain retractors, may not be generalizable to centers that employ more conventional approaches with greater cerebellar retraction, the magnitude of benefit observed here may not translate directly to other settings.

Beyond the primary antiemetic effects, granisetron-treated patients demonstrated significantly better early mobilization at six hours postoperatively and higher oral intake at breakfast on POD 1. These secondary benefits are clinically relevant in MVD, where early mobilization is important for patient comfort and recovery. These functional improvements may partly reflect better early PONV control; however, they could also be influenced by other factors such as postoperative pain, dizziness, nursing practices, and recovery protocols, and a causal link to PONV reduction cannot be established from this study.

Limitations

Our study has several limitations. The relatively small sample size (40 patients) limits the generalizability of our findings and the precision of the effect estimates. This retrospective, single-center design, with treatment allocation based on temporal changes in the treatment protocol, introduced potential confounding. Although baseline differences existed between the groups, these factors are unlikely to have substantially influenced the primary results. Furthermore, the small sample size precluded formal multivariate analysis to adjust for potential confounders such as age, diagnosis, and surgical duration, all of which may independently influence PONV outcomes. As is inherent to quasi-experimental before-after designs, temporal confounding cannot be fully excluded. The concurrent reduction in intraoperative fentanyl use in the granisetron group, arising from a national supply restriction beginning in December 2024, represents one such temporal variable; however, previous studies have demonstrated that intraoperative opioid administration does not significantly contribute to PONV [16,17], mitigating the likely impact of this confounder.

Additionally, outcome assessments relied on nursing documentation, which may introduce subjective variability. Adverse events were ascertained retrospectively from routine clinical documentation rather than by prospective systematic assessment. As a non-randomized study, residual confounding from unmeasured variables cannot be excluded, and the highly standardized, single-surgeon technique limits generalizability to other centers. We did not measure CSF serotonin levels, which would have provided direct evidence supporting the brainstem serotonin-release hypothesis proposed in this discussion. Furthermore, we did not assess postoperative pain levels, which may have influenced secondary outcomes, including mobilization and oral intake. Future prospective studies incorporating CSF biomarker analysis and randomized comparisons of multimodal antiemetics would further elucidate the relative contributions of serotonergic and non-serotonergic pathways to PONV after MVD.

## Conclusions

In this exploratory, single-center, quasi-experimental before-after study of 40 patients, prophylactic granisetron was associated with an RRR of 56% and an ARR of 45% (NNT ≈2.2) for moderate-to-severe PONV following MVD, consistent with a substantial serotonergic contribution to PONV in this setting. The residual PONV rate of 35% despite granisetron prophylaxis suggests that non-serotonergic emetic mechanisms remain unaddressed. Given the small sample size, single-center design, baseline differences between groups, and potential for temporal confounding, these findings should be interpreted as hypothesis-generating rather than confirmatory. Nonetheless, they support the evaluation of multimodal antiemetic strategies and may inform the design of future adequately powered, multicenter randomized studies on optimal antiemetic strategies for MVD.
